# Sources of information on surgical options for patients undergoing antireflux surgery: a single-center questionnaire-based survey

**DOI:** 10.1186/s12876-023-02712-8

**Published:** 2023-03-14

**Authors:** Ningying Ruan, Hao Chen, Zhifei Wang, Jinlei Mao, Jianfu Xia, Fei Zhao, Ting Zhang

**Affiliations:** 1 Hangzhou Medical College, Hangzhou, China; 2 Zhejiang Chinese Medical University, Hangzhou, China; 3 General Surgery, Cancer Center, Department of Hepatobiliary & Pancreatic Surgery and Minimally Invasive Surgery, Zhejiang Provincial People’s Hospital, Affiliated People’s Hospital, Hangzhou Medical College, Hangzhou, Zhejiang China; 4 Wenzhou Central Hospital, Wenzhou, China; 5 Zhejiang Provincial People’s Hospital, Hangzhou, China

**Keywords:** GERD patients, Antireflux surgery, Information source, Media

## Abstract

**Background:**

To explore the sources of information on antireflux surgery for patients undergoing this surgery in China.

**Methods:**

Patients who underwent antireflux surgery in the Gastroesophageal Reflux Center of the Zhejiang Provincial People's Hospital from January 2016 to June 2021 were selected as survey subjects, and a questionnaire survey was conducted by telephone.

**Results:**

A total of 358 questionnaires were distributed, and 320 valid questionnaires were recovered, yielding a 89.4% completion rate. Among patients' sources of information about antireflux surgery, the media was the primary source (33.8%) followed by recommendations from relatives or friends (27.8%), referrals from physicians (23.4%) and other sources (15.0%). Patients of different ages and educational levels have different sources for obtaining information about the procedure. Most of the information on surgery for patients aged 20 to 49 years was derived from recommendations from friends or relatives, whereas most of the information on surgery for patients aged 50 to 80 years was obtained from the media. Most of the information on surgery for patients with a primary school education or less was derived from physicians' recommendations, whereas most of the information for patients with a junior secondary school education or higher was obtained from the media. The recommendation of patients for surgery varied among the different departments (X^2^ = 36.011, *p* < 0.001), and a two-to-two comparison found that the recommended rates for cardiology and gastroenterology differed from the rates of other groups (*p* < 0.001, Table 2).

**Conclusions:**

The results of this investigation show that a large number of patients who underwent antireflux surgery learned about the operation through the media and recommendations from relatives or friends rather than physicians at the hospital. Notably, physicians specializing in GERD need to increase their knowledge of the disease and surgical treatment options to provide correct medical information to patients and to conduct media campaigns.

## Introduction

Gastroesophageal reflux (GERD) is a disorder in which gastric contents reflux into the oesophagus, causing uncomfortable symptoms and/or complications [1]. Most GERD patients exhibit typical clinical manifestations, such as heartburn, acid reflux, and belching. These symptoms can be reduced by adjusting lifestyle and diet and improved by taking medications, such as PPIs.

Antireflux surgery is an effective alternative to drug treatment [2]. Surgery is indicated for some patients who need long-term maintenance therapy with PPIs and whose efficacy is insufficient as well as some patients with secondary complications of GERD or extraoesophageal symptoms given that surgery can effectively improve reflux symptoms and reduce the need for medication [3]. In China, many patients are not able to access information related to antireflux surgery and are therefore deprived of the benefits of this surgery. The purpose of this paper is to analyse the sources of surgical information of patients undergoing antireflux surgery, identify existing problems, and provide more patients suffering from GERD who would benefit from surgery with information on antireflux surgery given its ability to potentially cure this condition.

## Methods

### Study population

Patients who underwent antireflux surgery at the GERD centre of the Zhejiang Provincial People's Hospital were selected for investigation from January 2016 to June 2021, and all patients were included in the study on a voluntary basis. Foreign patients, emergency cases, patients in need of additional treatments and those with mental disorders were excluded from the study. A questionnaire survey was conducted by telephone. In total, 358 questionnaires were distributed, and 320 valid questionnaires were returned, yielding an 89.4% response rate.

### Hospital characteristics and referral patterns in China

Our hospital is a provincial-level tertiary hospital. Our GERD treatment centre is nationally recognized and performs a high volume of antireflux surgery in China, with 100 to 150 cases of antireflux surgery performed each year via either laparoscopic surgery or robotic surgery. In addition, it is a renowned and accredited training centre for antireflux surgery.

In China, there is no standardized referral system or pattern for family physicians or between big tertiary and primary hospitals. Most of the insurance relating to referral policy is from the government, yet the referral policy varies. In some places, patients with a referral can be covered 10% to 15% more compared with no referral. In contrast, there is no difference in other areas, or patients can still choose to go straight to a hospital without referral systems. Still, in some places, the insurance would not cover a patient’s expense at another hospital without a referral. However, no restrictions on referral will add burdens to the local government’s finance.

### Identification of indications for surgery

The basis for determining the indications for surgery in this article is similar to the Chinese Consensus on Multidisciplinary Management of Gastroesophageal Reflux Disease [4]. Before the surgery, we assess the patient's symptoms and establish an indication using a myriad of tools, such as upper gastrointestinal contrast, gastroscopy, 24-h oesophageal pH monitoring, and oesophageal manometry.

### The questionnaire

The contents of the survey included the patient's gender, age, education, symptoms of the first visit, department of the first visit, medical history, whether they received regular drug treatment, satisfaction with surgery, symptom improvement, and access to information.

### Statistical analysis

Excel and IBM SPSS Statistics for Windows (version 23.0) were used to statistically analyse the questionnaires received. Pearson's chi-squared test was used to compare multiple independent groups. A p value of less than 0.05 was considered significant.

## Results

### Basic information

**Fig. 1:**
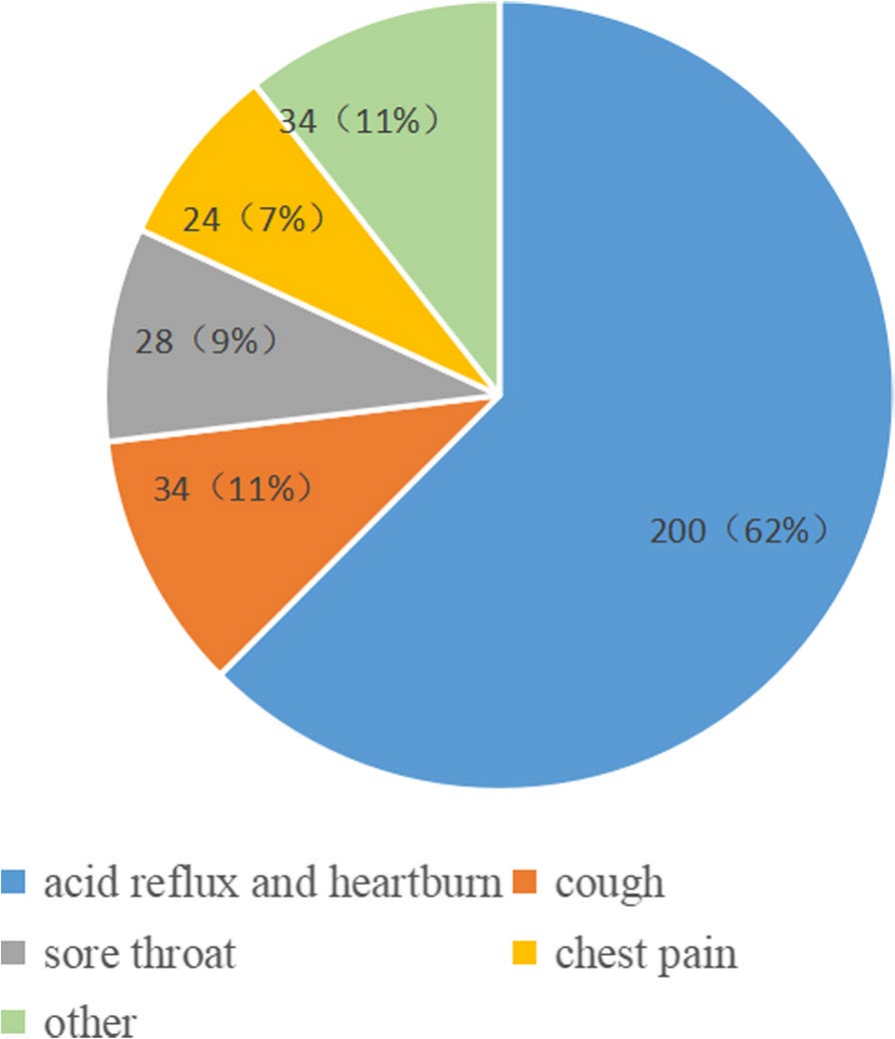
Percentage of patients with major symptoms

Of the 320 patients who participated in this questionnaire survey, 166 (51.9%) were female, and 154 (48.1%) were male. Approximately half of the patients were aged between 50 and 64 years old, and the distribution of patients' education was roughly the same. Specific information on age and education distribution is presented in Table 1. The main symptom observed in 62% of patients was acid reflux and heartburn, and other patients atypical extraoesophageal symptoms, such as cough and chest pain (Fig. 1). The patients exhibited the following durations of GERD medical histories before surgery: 0–2 years in 205 (64.1%) patients, 3–4 years in 46 (14.4%) patients, and 5–6 years in 26 (8.1%) patients, 15 (4.7%) patients were 7–8 years, and more than 8 years in 28 (8.8%) patients. A total of 212 (66.3%) patients were satisfied with the surgical results, 66 (20.6%) patients were basically satisfied, 41 (12.8%) were dissatisfied, and one patient did not answer the question. In total, 135 (42.2%) patients had complete improvement in postoperative symptoms. A total of 151 (47.2%) patients exhibited basic improvement in postoperative symptoms, and 34 (10.6%) patients reported no improvements in postoperative symptoms.

**Table 1:** Sources of information on surgery for patients of different age groups and educational backgrounds

Classification basis	Grouping	Number	Access			
			Media	Introduced by friends, etc	Physician's referral	Others
Age	20 ~ 34	28	6	13	6	3
			(21.4%)	(46.4%)	(21.4%)	(19.7%)
	35 ~ 49	86	27	32	18	9
			(31.4%)	(37.2%)	(20.9%)	(10.5%)
	50 ~ 64	156	53	36	37	30
			(34.%)	(23.1%)	(23.7%)	(19.2%)
	65 ~ 80	50	22	8	14	6
			(44.0%)	(16.0%)	(28.0%)	(12.0%)
Academic qualifications	Primary school and below	72	13	18	26	15
			(18.1%)	(25.0%)	(36.1%)	(20.8%)
	Junior high school	101	39	24	23	15
			(38.6%)	(23.8%)	(22.8%)	(14.9%)
	Senior high school	66	24	18	13	11
			(36.4%)	(27.3%)	(19.7%)	(16.7%)
	University and above	81	32	29	13	7
			(39.5%)	(35.8%)	(16.0%)	(8.6%)

### Analysis of information sources for patients to learn about surgical options

**Fig. 2:**
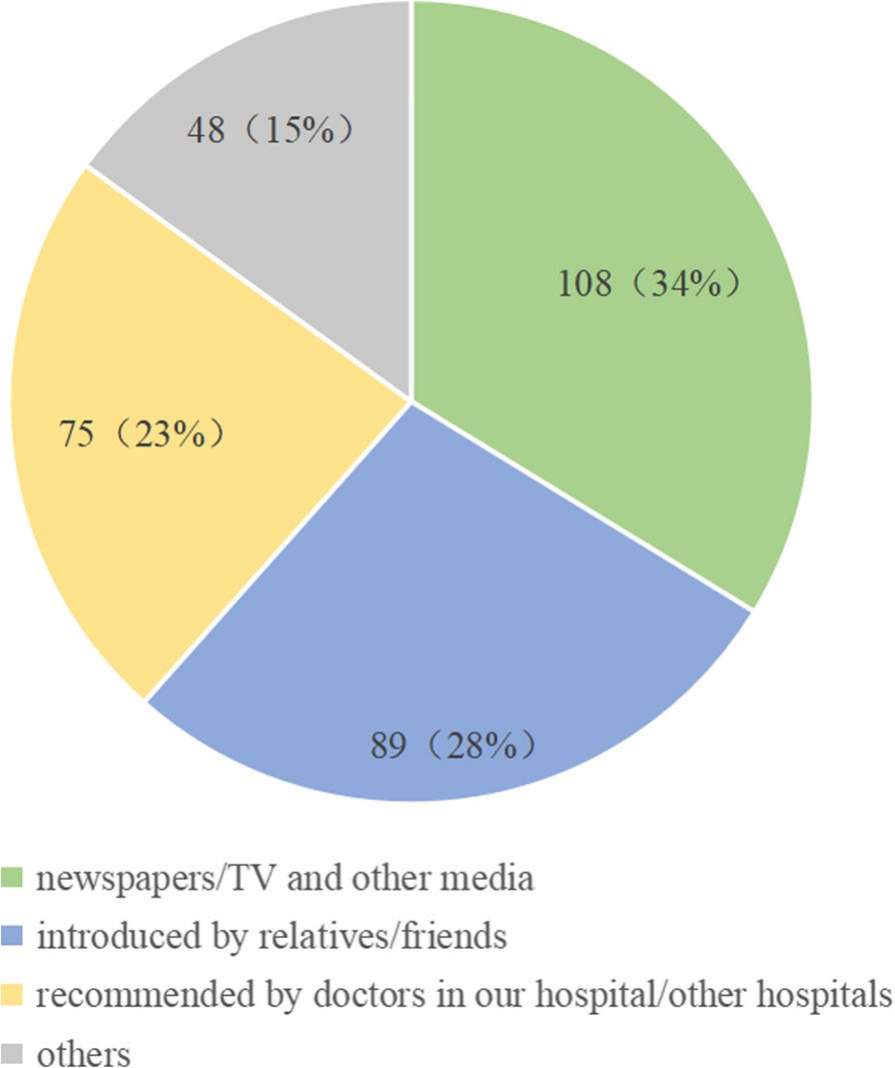
Patients' surgical information sources

Before undergoing surgical treatment, numerous patients learned about antireflux surgery through the media followed by recommendations from friends or relatives and referrals by physicians (Fig. 2). For the patients who obtained the information from media, information was obtained from newspapers (57.4%), TV programs (25.9%), and other sources (16.7%). Among the patients referred to by physicians at our hospital or other hospitals for surgical treatment, 34 (45.3%) were referred from the gastroenterology department, 7 (9.3%) from the pulmonary department, 9 (12.0%) from the otolaryngology department, and 17 (22.7%) from the cardiology department. The remaining 8 (10.7%) patients were referred from other departments.

### Analysis of information sources for patients in different age groups and with educational backgrounds

The results showed that most of the information on surgery for patients aged 20 to 49 years was derived from recommendations from friends or relatives, whereas most of the information on surgery for patients aged 50 to 80 years was obtained from the media (Table 1). Most of the information on surgery for patients with primary school education or less was obtained from physicians' recommendations, whereas most of the information for patients with junior secondary school education or higher was obtained from the media (Table 1).

### Analysis of patient referral for surgery by physicians in different departments

We categorized patients according to the different symptoms that would lead them to seek treatment at different departments and analysed whether the physicians in the different departments recommended the patients for surgery. The recommendation of patients for surgery varied between the different departments (X^2^ = 36.011, *p* < 0.001). A two-to-two comparison found that the recommended rates for cardiology and gastroenterology differed from those of other groups (*p* < 0.001, Table 2).

**Table 2:** The situation of physicians in different departments recommending patients for surgical treatment

Department type	Recommendation		
	Yes (%)	No (%)	Total
Gastroenterology	34 (17.0)	166 (83.0)	200
Pulmonary	7 (20.6)	27 (79.4)	34
Otolaryngology	9 (32.1)	19 (67.9)	28
Cardiology	17 (70.8)	7 (29.2)	24

## Discussion

From the data, we found that the first source of information for patients about antireflux surgery was the media (34%) with newspaper media (57.4%) dominating; the second most common source of information included recommendations from others (including relatives, friends, and patients) (28%). Only a small proportion of patients received information about the surgery from physicians (23%).

On the one hand, the majority of patients' information about surgery is obtained from the media and other people rather than from physicians. This finding reveals a problem with the physician's ability to distribute information. In general, patients only seek further information on GERD treatments from the media or from others after learning that they have the disease and that their current treatment is not working well. The fact that the majority of patients do not receive information about surgery from physicians means that they have not been informed about the procedure from their physicians during their previous visits and have only learned about this treatment option through the media or from others.

We categorized the patients according to the different symptoms that would lead them to seek treatment at different departments and analysed whether the physicians in the different departments recommended the patients for surgery. We found that cardiologists were more likely to recommend surgery to patients, whereas gastroenterologists, who knew the most about the disease, were less likely to recommend patients for surgery. In addition, given that antireflux surgery was only first described in Chinese surgical textbooks in 2018 [5], some physicians are unaware of the procedure. Only a small number of surgeons know how to perform it, which leads to a lack of understanding of the procedure on the part of physicians. Thus, some patients who do not respond to medication and are eligible for surgery are missing out on the possibility for surgical treatment. These patients continue to suffer from the disease and spend a lot of time and money [6, 7], especially patients who present with extraoesophageal symptoms [8].

On the other hand, the finding that the media is the first source of information for most patients shows that the media is a significant factor that affects every area of human life from education to health care [9]. Accurate and objective media campaigns help patients learn more about health care [10–12]. Among them, based on our analysis of the source of surgical information for patients with different age groups and academic qualifications, it was revealed that patients aged 50 years and above with a junior high school education obtain most of their surgical information from the media, including news reports and TV interviews performed by the hospital. However, as mentioned above, physicians should provide sufficient awareness about the disease and surgical protocols, but the current level of physician-based information is insufficient. Physician-based information should be popularized through the media to reach more patients.

The patients in this study were all indicated for surgery, as demonstrated by the preoperative workup, and symptoms were resolved in 89.4% of patients postoperatively, which is similar to that reported in the literature [13, 14]. Of course, many patients referred to us were not diagnosed with GERD or did not meet the criteria of surgical treatment (regardless of whether these patients received information from friends and relatives or the media).

Since Nissen performed the first fundoplication in 1936, fundoplication has become the classic procedure for GERD and has achieved very satisfactory results. This procedure can eradicate acid reflux and heartburn in approximately 90% of GERD cases [15] and is considered the most suitable procedure for most GERD patients except for those with a shortened oesophagus [16]. The Rossetti method (modified Nissen), the Toupet method (270° fold), and the Dor method (180° fold) are also widely used. However, these procedures are associated with complications, such as dysphagia, flatulence syndrome, and diarrhoea, and require a rigorous preoperative assessment by physicians for the development of an individualized surgical treatment plan. We believe that for those patients with GERD who meet the indications for surgery, the surgical treatment plan should be developed by the physician to avoid the possibility that the patient's symptoms have not improved significantly after the second or even multiple visits, which requires gastroenterology, cardiology, respiratory medicine and several specialists related to GERD and related complications to deepen their understanding of the disease and surgical treatment, and recommend patients who meet the surgical indications to surgical treatment promptly.

In addition, media can effectively fill the physician's information gap to a certain extent. There is a multidisciplinary consensus on the diagnosis and treatment of GERD in China [4]. However, this consensus is not written in a style that is accessible to patients. The guidelines are published in academic journals that are not regularly accessible to patients, so patients tend to obtain information from the media. This finding again raises another problem. Specifically, the quality of the current Chinese media varies, and unprofessional media information can mislead patients by providing incorrect medical information. Moreover, many physicians do not read the published guidelines. Interestingly, physicians can use social media, such as TikTok and Kuaishou, to deliver medical knowledge with limited restrictions, and these information platforms have become very popular in China. However, more regulations should implemented to encourage media outlets to provide more patient education and authentic information.

Our hospital is a provincial tertiary care hospital and one of the largest gastroesophageal reflux centres in China. The number of surgeries performed at this centre has been increasing yearly with an annual volume of more than 100 cases in recent years. Thus, this survey is somewhat representative. However, there are limitations due to the varying number of patients who undergo antireflux surgery at our hospital each year. In addition, the questionnaire relies on the subjective feelings of patients, so some data lack a certain degree of objectivity.

## Conclusion

The results of this investigation show that a large number of patients who underwent antireflux surgery learned about the operation through the media and recommendations from relatives or friends rather than physicians at the hospital. Notably, physicians specializing in GERD need to increase their knowledge of the disease and surgical treatment options to provide correct medical information to patients and to conduct media campaigns.

## Data Availability

The datasets used and/or analysed during the current study are available from the corresponding author on reasonable request.

## Abbreviations

GERD: Gastroesophageal reflux disease
PPIs: Proton pump inhibitors
